# The role of minimally invasive surgery for diagnosis and staging in paediatric surgical oncology

**DOI:** 10.3332/ecancer.2025.2032

**Published:** 2025-11-13

**Authors:** Tristan Boam, Diego Aspiazu Salinas

**Affiliations:** 1 Department of Paediatric Surgery, Children’s Health Ireland at Crumlin Hospital, Dublin, D12 N512 Ireland; 2 Department of Paediatric Surgery, Clínica San Felipe, Clinica Angloamericana, Clinica Internacional, Lima 15073, Perú

**Keywords:** minimally invasive surgery, paediatric oncology, diagnosis, staging, biopsy, indocyanine green

## Abstract

Minimally invasive surgery (MIS) has become increasingly important in paediatric surgical oncology for the diagnosis and staging of solid tumours, due to its advantages in reducing morbidity, pain and hospitalisation times compared to traditional open surgery. While ultrasound-guided core needle biopsy (USCNB) typically remains the primary method for tissue sampling, MIS becomes essential in cases where USCNB is impractical or ineffective, such as with inaccessible tumour locations or where detailed staging information is required. Recent studies highlight the effectiveness of MIS in obtaining high-quality biopsy samples in neuroblastoma, thoracic tumours, hepatoblastoma and rhabdomyosarcoma, frequently outperforming open surgical methods regarding sample adequacy and complication rates. Video-assisted thoracoscopic surgery has demonstrated particular efficacy with minimal complications across various thoracic malignancies. Additionally, laparoscopic and robotic approaches for retroperitoneal lymph node dissection in rhabdomyosarcoma have proven beneficial by significantly reducing postoperative complications and hospital stays compared to open methods. Innovative adjunct technologies such as indocyanine green (ICG) fluorescence imaging have further advanced MIS by providing superior visualisation of tumour margins, metastases and lymphatic structures, enhancing the precision and safety of procedures. Overall, the integration of MIS techniques, supported by advanced imaging methods like ICG, represents a significant advancement in paediatric oncology, offering reliable diagnostic and staging options with reduced patient morbidity. These approaches provide critical clinical advantages, positioning MIS as an essential component of contemporary paediatric surgical oncology practice.

## Introduction

Minimally invasive surgery (MIS) is increasingly integrated into paediatric surgical oncology, providing significant benefits such as reduced pain, shorter hospital stays and quicker recovery compared to traditional open surgery. Ultrasound-guided core needle biopsy (USCNB) is often the procedure of choice for tissue diagnosis; however, MIS is an important option if the tumour is not accessible with this technique. This guideline reviews the role of MIS specifically for biopsy and staging, addressing its utility in various paediatric solid tumours.

## MIS indications for diagnosis and staging

### Neuroblastoma

A European retrospective study compared four biopsy methods for neuroblastoma: USCNB (most frequently used, 37.5%), laparoscopic-assisted core needle biopsy (39.6%), open incisional biopsy (12.5%) and minimally invasive thoracoscopic/laparoscopic incisional biopsy (10.4%). Perioperative complications were rare overall, with one major complication (duodenal perforation) reported in the open biopsy group and minor complications conservatively managed in the USCNB and laparoscopic-assisted core needle biopsy. MIS techniques demonstrated effectiveness in providing adequate tissue for histopathological and molecular analyses, achieving a 100% success rate in all modalities. In contrast, open incisional biopsies had the highest inadequacy rate at 16.7%, followed by USCNB at 11%, although these differences were not statistically significant [1].

Another case report highlighted a potential role for MIS staging, describing the use of robotic-assisted lymph-node dissection for an L1 neuroblastoma after adrenalectomy [2].

### Thoracic tumours

In a single institution series, video-assisted thoracoscopic surgery (VATS) demonstrated high effectiveness and low morbidity in obtaining biopsies for various thoracic tumours, including neuroblastoma, ganglioneuroma, germ cell tumours, lymphoma and thymic tumours. There were low complication (7%) and conversion rates (2.3%), supporting its feasibility for biopsy [3].

### Wilms tumour

A Children’s Cancer and Leukaemia Group and the International Society of Paediatric Oncology (SIOP) report on Wilms tumour biopsy experience from the SIOP 2001 trial showed that the majority of biopsies are performed by USCNB, with a 6.5% rate of non-diagnostic sampling. No biopsies were performed by MIS; 10 were open incisional biopsies. In the majority of cases, biopsy for Wilms tumour is generally unnecessary and is typically reserved for cases with imaging features and clinical factors that raise suspicion of other malignancies such as clear cell sarcoma, rhabdoid tumour or renal cell carcinoma [4]. USCNB is preferred in these scenarios, as the tumours are often readily accessible and it is likely to be associated with less risk of spillage, upstaging and local recurrence compared to open and MIS; notably, the UKW3 trial did not associate USCNB with increased local recurrence and its use should not mandate upstaging of the disease [5].

### Hepatoblastoma

MIS may have a potential role for staging in hepatoblastoma, in scenarios where cross-sectional imaging is equivocal or unavailable; laparoscopy may have utility in confirming involved liver segments and extra-hepatic metastases.

A multicentre retrospective study examining biopsy methods for hepatoblastoma concluded that USCNB was associated with significantly reduced bleeding complications compared to MIS or open methods, supporting USCNB as the preferred initial diagnostic modality [6].

### Rhabdomyosarcoma

In a small case series of laparoscopic retroperitoneal lymph node dissection (RPLND) for paratesticular rhabdomyosarcoma, the authors found, while technically challenging, the technique is associated with reduced morbidity compared to open approaches [7].

A further comparative retrospective study demonstrated single-site retroperitoneoscopic RPLND as equally effective with significantly reduced postoperative analgesia hospital stay compared to traditional laparoscopic techniques [8].

Robotic-assisted RPLND was evaluated in an adolescent series, demonstrating comparable lymph node yield and minimal complications [9].

### Lung metastases

In a 2023 report, VATS showed efficacy for identification and excisional biopsy of pulmonary metastases, especially when combined with pre-operative computed tomography-guided coil localisation. This combination significantly improved surgical accuracy, ensuring complete resection with maximum sparing of normal lung tissue [10]. Another study indicated that VATS pulmonary metastasectomy had comparable outcomes to open thoracotomy in cases of oligometastatic osteosarcoma [11].

## Summary of MIS indications

- For tumour biopsy where USCNB is not suitable or unavailable.
- Lymph node sampling and dissection.
- VATS excision biopsy of lung nodules/metastases.
- MIS staging if cross-sectional imaging is equivocal or unavailable.

## MIS contraindications for diagnosis and staging

Contraindications to MIS are generally few, especially when it is used primarily for diagnostic and staging purposes. However, the role of MIS for biopsy does have important considerations for a number of scenarios in paediatric surgical oncology. Biopsies should only be taken when necessary, if the diagnosis cannot be reached by clinical or non-invasive means. When they are necessary, biopsies should be performed by the least invasive route. With modern imaging techniques and high-resolution ultrasound, USCNB is often the procedure of choice. If USCNB is unavailable, for example, in resource-poor environments and developing nations, MIS is likely the next-best option for staging, diagnosis and biopsy, as it makes use of transferable skills and widely available equipment.

MIS should also be avoided in cases where port access is made unsafe by factors such as dense abdominal adhesions, distended bowel loops or in patients where pneumoperitoneum or pneumothorax induction could lead to respiratory compromise.

## Summary of MIS contraindications

- If an alternative to biopsy is available.
- If USCNB is available.
- In patients with significant cardiac/respiratory compromise.
- In patients where port access is unsafe, e.g., dense adhesions.

## Surgical approach

### Patient position

Patient positioning depends on tumour location; abdominal tumours usually require a supine or slightly lateral position, while VATS procedures use lateral decubitus or modified prone positions.

### Trocar sites

Trocar placement also depends on the tumour location. Optimal placement for biopsies should focus on minimising the risk of tumour spillage and safe extraction of the tumour sample.

### Surgical technique, tips and pitfalls

For MIS biopsy, several key techniques are necessary to ensure sample quality and procedural safety. Cold endoscopic scissors are recommended to prevent thermal damage to biopsy samples that can occur with diathermy use. After sampling, placing the biopsy specimen into an endoscopic retrieval bag reduces the risk of spillage and tumour seeding.

Once the sample is taken, diathermy should remain readily available to manage potential bleeding. Additional haemostatic adjuncts such as Tisseal™ (fibrin sealant) and TachoSil™ (fibrin-coated collagen patch) are beneficial for achieving rapid and effective haemostasis.

In certain scenarios, a laparoscopic-assisted core needle biopsy may be utilised; MIS is beneficial for moving obstacles to biopsy (e.g., overlying bowel loops) and the needle can be guided under direct vision.

During VATS lung/pleural biopsy procedures, energy devices such as LigaSure™ facilitate efficient vessel sealing and tissue dissection. Additionally, the authors favour Endo GIA™ staples to effectively manage lung parenchyma.

## Use of indocyanine green (ICG) fluorescence for diagnosis and staging

ICG has emerged as a valuable tool in paediatric surgical oncology; its ability to fluoresce under near-infrared light allows it to accurately delineate tumour margins, detect hidden metastases and facilitate lymphatic mapping intraoperatively. The technology complements conventional imaging techniques and contributes to a more precise and conservative surgery, with the potential to improve oncological and functional results in children and adolescents with solid neoplasms.

### ICG in hepatoblastoma

ICG has been used in many instances for assisting both primary tumour resection and the identification of metastases, aiding minimally invasive pulmonary metastasectomy [12].

A South Korean study involving 17 patients undergoing ICG fluorescence-guided radical hepatoblastoma resection demonstrated its precision in delineating tumour margins and its potential feasibility for detecting tumour spread and lesions not visible on conventional imaging [13].

### ICG for pulmonary metastases

A Japanese study showed that ICG facilitated intraoperative detection of pulmonary micro-metastases that were not identified by palpation or preoperative imaging. A total of 250 fluorescent nodules were resected, some as small as 62 µm, suggesting a significant improvement in detection thresholds [14].

### Identification of occult lesions

A study from China involving 16 patients revealed that the use of ICG enabled the identification of and resection of additional lesions that were not detected in preoperative imaging, achieving negative surgical margins in all cases [15].

### Sentinel lymph node mapping with ICG

A prospective study evaluated the use of ICG in combination with technetium-99m for sentinel lymph node biopsies in paediatric and adolescent patients. ICG fluorescence demonstrated 100% sensitivity in identifying the sentinel lymph node, with no adverse events reported [16].

### Wilms tumour lymph node sampling with ICG

Lymph node sampling is a critical step in Wilms tumour nephrectomy. Recent publications have highlighted the utility of intraparenchymal injection of ICG to aid lymph node identification during MIS nephrectomy [17, 18]. A recent single-centre study of MIS nephrectomies comparing 7 patients that received ICG-guided lymph node sampling to 18 controls suggested that the technique is safe, feasible and may improve the total lymph node yield [19].

## Conclusion

MIS remains an essential tool for the diagnosis and staging of paediatric solid tumours. Its role will likely evolve for procedures that once mandated an open approach, such as RPLND, enhanced by newer robotic and image-guided techniques, such as ICG fluorescence. Its current mainstay is that of a valuable option should the more common USCNB be unavailable or impossible in challenging scenarios.

## Conflicts of interest

None to declare.

## Funding

No specific funding received.

## References

[1] Paraboschi I, Bolognesi E, Giannettoni A (2021). The role of biopsy in the workup of patients with neuroblastoma: comparison of the incidence of surgical complications and the diagnostic reliability of diverse techniques. Children.

[2] Uwaydah NI, Jones A, Elkaissi M (2014). Pediatric robot-assisted laparoscopic radical adrenalectomy and lymph-node dissection for neuroblastoma in a 15-month-old. J Robot Surg.

[3] Riccipetitoni G, Bertozzi M, Gazzaneo M (2021). The role of video-assisted thoracoscopic surgery in pediatric oncology: single-center experience and review of the literature. Front Pediatr.

[4] Jackson TJ, Williams RD, Brok J (2019). The diagnostic accuracy and clinical utility of pediatric renal tumor biopsy: report of the UK experience in the SIOP UK WT 2001 trial. Pediatr Blood Cancer.

[5] Irtan S, Jitlal M, Bate J (2015). Risk factors for local recurrence in Wilms tumour and the potential influence of biopsy - the United Kingdom experience. Eur J Cancer.

[6] Weldon CB, Madenci AL, Tiao GM (2020). Evaluation of the diagnostic biopsy approach for children with hepatoblastoma: a report from the children’s oncology group AHEP 0731 liver tumor committee. J Pediatr Surg.

[7] Tomaszewski JJ, Sweeney DD, Kavoussi LR (2010). Laparoscopic retroperitoneal lymph node dissection for high-risk pediatric patients with paratesticular rhabdomyosarcoma. J Endourol.

[8] El-Gohary Y, Mansfield S, Talbot L (2020). Single-site retroperitoneoscopy in pediatric metastatic lymphadenopathy. J Pediatr Surg.

[9] Cost NG, DaJusta DG, Granberg CF (2012). Robot-assisted laparoscopic retroperitoneal lymph node dissection in an adolescent population. J Endourol.

[10] Warmann SW, Lieber J, Schaefer JF (2023). Thoracoscopic resection of lung nodules following CT-guided labeling in children and adolescents with solid tumors. Children.

[11] Lautz TB, Farooqui Z, Jenkins T (2021). Thoracoscopy vs thoracotomy for the management of metastatic osteosarcoma: a Pediatric Surgical Oncology Research Collaborative Study. Int J Cancer.

[12] Hiyama E (2021). Fluorescence image-guided navigation surgery using indocyanine green for hepatoblastoma. Child (Basel, Switzerland).

[13] Cho YJ, Namgoong JM, Kwon HH (2021). The advantages of indocyanine green fluorescence imaging in detecting and treating pediatric hepatoblastoma: a preliminary experience. Front Pediatr.

[14] Yamada Y, Ohno M, Fujino A (2019). Fluorescence-guided surgery for hepatoblastoma with indocyanine green. Cancers (Basel).

[15] Shen Y, Zheng M, Li J (2022). Clinical application of indocyanine green fluorescence imaging in the resection of hepatoblastoma: a single institution’s experiences. Front Surg.

[16] Pio L, Richard C, Zaghloul T (2024). Sentinel lymph node mapping with Indocyanine green fluorescence (ICG) for pediatric and adolescent tumors: a prospective observational study. Sci Rep.

[17] Abdelhafeez AH, Davidoff AM, Murphy AJ (2022). Fluorescence-guided lymph node sampling is feasible during up-front or delayed nephrectomy for Wilms tumor. J Pediatr Surg.

[18] Pachl MJ (2021). Fluorescent guided lymph node harvest in laparoscopic Wilms nephroureterectomy. Urology.

[19] Roberts R, Pachl M (2024). Intraparenchymal indocyanine green use improves nodal yield during minimally invasive tumor nephrectomy in children. J Laparoendosc Adv Surg Tech A.

